# Use of aerosol route to fabricate positively charged Au/Fe_3_O_4_ Janus nanoparticles as multifunctional nanoplatforms

**DOI:** 10.1038/srep35104

**Published:** 2016-10-07

**Authors:** Jeong Hoon Byeon, Jae Hong Park

**Affiliations:** 1 School of Mechanical Engineering, Yeungnam University, Gyeongsan 38541, Republic of Korea; 2 School of Health Sciences, Purdue University, IN 47907, United States

## Abstract

Gold (Au)-decorated iron oxide (Fe_3_O_4_), Au/Fe_3_O_4_, Janus nanoparticles were fabricated via the continuous route for aerosol Au incorporation with Fe_3_O_4_ domains synthesized in an aqueous medium as multifunctional nanoplatforms. The fabricated nanoparticles were subsequently exposed to 185-nm UV light to generate positive charges on Au surfaces, and their activities were tested in computed tomography (CT) and magnetic resonance (MR) imaging, gene-delivery and photothermal therapy. No additional polymeric coatings of the Janus particles also had a unique ability to suppress inflammatory responses in macrophages challenged with lipopolysaccharide, which may be due to the absence of amine groups.

Heterogeneous nanostructures consisting of different nanoscale components that may introduce multifunctionality have received a great deal of attention in various applications, such as energy production/storage, catalysis, and biomedical applications. Because of the synergetic or complementary effects from their unique structural and interfacial properties, hetero-nanostructures can derive favorable physicochemical characteristics that can break through the current technical limits to the realistic employment of monocomponent nanomaterials for future nanotechnology^1^. As one of the representative hetero-nanostructures, Janus or dumbbell-like particles consisting of different components have been considered favorably due to their multifunctionality^2^. For biomedical applications, in particular, the use of hetero-nanostructures as probes via the incorporation of two or more functional materials may introduce dual- or multi-modal responses to enable therapeutics and diagnostics to be performed simultaneously^3^.

Gold (Au)-decorated iron oxide (Fe_3_O_4_), Au/Fe_3_O_4_, nanostructures have received great attention as representative hetero-nanostructures because of their biocompatibility and multifunctionality. The incorporation of Au and Fe_3_O_4_ components can basically bring together plasmonic and magnetic properties, and these components are representative biocompatible metallic components to be employed in biomedical applications^4^,^5^. Specifically, various Au nanostructures have recently been adopted in chemo- or photo-thermal therapies because of their surface plasmon resonance (SPR) heating when they are placed on visible or near-infrared (NIR) irradiation^5^,^6^,^7^. Particularly, NIR has high transmission efficiency into water or hemoglobin; thus it may be more suitable to penetrate into deep tissues to treat or kill cancer cells using a non-invasive method. The high thermal energies from the irradiation are capable of treating or killing cancer cells owing to thermoresponsive drug release and the hyperthermic effect^7^.

Numerous approaches have been proposed for synthesizing functional nanomaterials that mainly consist of Au and Fe_3_O_4_ nanoparticles^8^,^9^. More recently, gold-iron oxide hybrid nanomaterials have been further considered to create multimodal theranostic nanoplatforms for biomedical applications using multiple wet chemical processes^10^,^11^,^12^. Template- or seed-mediated chemical approaches, where preformed metallic particles are used as templates or seeds for growing other metallic components on their surfaces, are well known for synthesizing hetero-nanostructures without forming monometallic particles. However, incorporating quantitatively different metallic components to form hetero-nanostructures is challenging, because most approaches consist of tedious multi-step wet chemical processes (which can lead to the generation of unwanted products), and this also requires additional cationic coating on the surfaces for targeting and/or binding purposes^13^,^14^. Moreover, to form metallic hetero-nanostructures in a chemical bath, a reducing agent (mostly toxic) converts metal ions to metal atoms; however, this may introduce complexities to the reaction and separation, and its use may be restricted in biomedical applications^4^. Despite the successful combination of the Au and Fe_3_O_4_ (or Fe_2_O_3_) components, the introduction of practical generalizable assembly strategies in a continuous manner is a huge challenge in the preparation of anti-inflammatory nanoplatforms without significant changes to the preparation system.

In this study, the potential use of positively charged Au/Fe_3_O_4_ Janus nanoparticles as multifunctional nanoplatforms via the single-pass aerosol route without multi-step chemical reactions and additional cationic coatings is discussed for use in computed tomography (CT)-magnetic resonance (MR) dual-mode imaging, gene-delivery, and photothermal therapy. Freshly produced aerosol Au nanoparticles were first injected into a collison atomizer filled with Fe_3_O_4_ nanoparticles to form hetero-droplets, and the droplets were successively injected into a diffusion dryer containing pelletized activated carbons and silica gels to extract solvent from the droplets, resulting in Au/Fe_3_O_4_ Janus nanoparticles. The Janus nanoparticles were then exposed to 185 nm UV radiation to eject some electrons from the surfaces of the nanoparticles in a single-pass configuration (Supplementary Fig. S1). Finally, the positively charged nanoparticles were electrostatically collected to evaluate their ability for CT-MR imaging, gene-delivery, and photothermal cancer cell killing [exposed to 705 nm wavelength laser light to inhibit adenosine triphosphate (ATP) production in cancer cells].

## Results and Discussion

**Figure 1:**
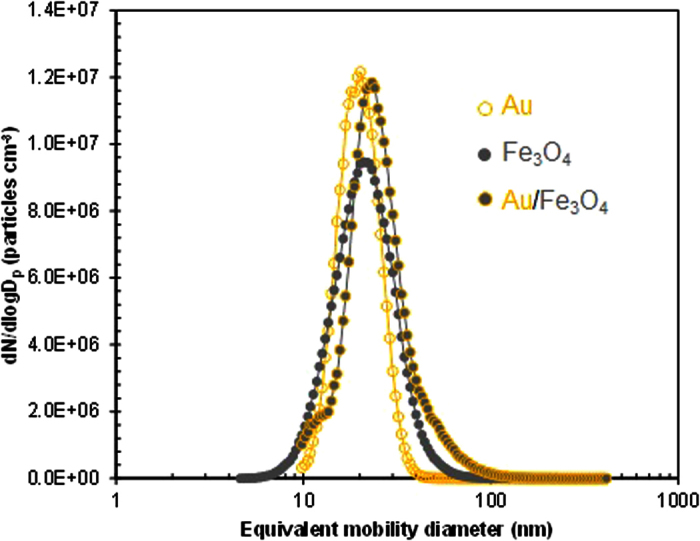
Size distributions of Au, Fe_3_O_4_, and Au/Fe_3_O_4_ nanoparticles in the aerosol state.

A gas temperature of approximately 6000 °C was generated between two cylindrical Au rods, and parts of the rods evaporated^15^. The duration of spark formation was approximately 1 ms, and the evaporated Au nucleated right after the spark channel by N_2_ gas flow, forming Au nanoparticles. The particle size distribution was analyzed using a scanning mobility particle sizer (3936, TSI, USA) to verify the concentration, mean diameter, and standard deviation. The measured concentration, diameter, and standard deviation of the Au particles were 3.45 × 10^6^ particles cm^−3^, 19.7 nm, and 1.28, respectively, as shown in Fig. 1. Au/Fe_3_O_4_ hetero-structures were fabricated via floating self-assembly, where Au nanoparticles were attached on Fe_3_O_4_ domains through single-pass collison atomization and solvent extraction, which were evaluated by a comparison of the size distributions (i.e., Fe_3_O_4_ and Au/Fe_3_O_4_ configurations, summarized in Supplementary Table S1) in the aerosol state. The particle concentration, diameter, and standard deviation of the Au/Fe_3_O_4_ particles were 4.10 × 10^6^ particles cm^−3^, 24.7 nm, and 1.49, respectively. Analogous data for the Fe_3_O_4_ particles were 3.22 × 10^6^ cm^−3^, 20.2 nm, and 1.46, respectively. Even though different size distributions were combined physically, there were no bi-mode (two individual peaks of Au and Fe_3_O_4_) characteristics in size distribution. The results showed only a new uni-mode size distribution and they were closer to those of the Au particles than those of the Fe_3_O_4_ domains, suggesting that the Au particles were attached well enough to the Fe_3_O_4_ domains to construct Au/Fe_3_O_4_ hetero-nanostructures.

**Figure 2:**
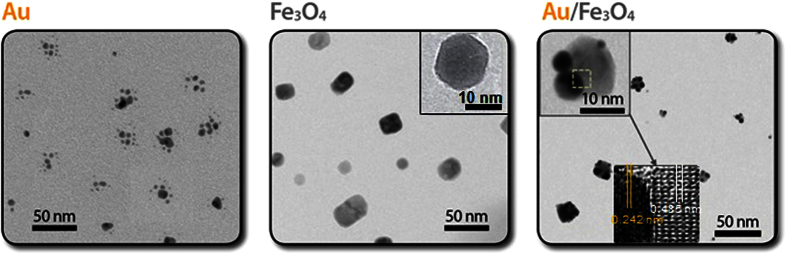
TEM observation results of Au, Fe_3_O_4_, and Au/Fe_3_O_4_ Janus (including high resolution images) nanoparticles. Floating self-assembly of Au and Fe_3_O_4_ nanoparticles induced random deposition of Au primary particles on Fe_3_O_4_ domains, resulting in formation of Janus nanoparticles.

The prepared particles (Au, Fe_3_O_4_, and Au/Fe_3_O_4_) in the aerosol state sampled on a carbon-coated copper grid were analyzed using a transmission electron microscope (TEM, JEM-3010, JEOL, Japan) (Fig. 2) to understand the formation of the Au/Fe_3_O_4_ hetero-nanostructures through the aerosol route. The particles were electrostatically deposited on the grid using a commercial aerosol collector (NPC-10, HCT, Korea). As shown in Fig. 2, the TEM observations suggest that the primary Au particles (about 3 nm mean diameter) were agglomerates, implying that the primary particles collided with each other after they formed near the spark. In the case of Fe_3_O_4_ domains, a square-like shape with a size of about 20 nm was observed, which was different from the spherical shape observed when Fe(CO)_5_ was used as the Fe-precursor^16^. For the Au/Fe_3_O_4_ configuration, a darker-contrast domain of spherical particles was exhibited in the Miller plane (111) owing to a 0.242-nm face-centered cubic Au lattice (inset), whereas lighter-contrast domains of the randomly deposited spherical particles were attributable to the Fe_3_O_4_ particles, showing the lattice spacing was about 0.485 nm (also see inset)^17^. Scanning electron microscope (SEM, NOVA nanoSEM, FEI, USA) images (Supplementary Fig. S2) further confirm the Au/Fe_3_O_4_ configuration. Thus, it was clearly observed that Au particles could be incorporated with Fe_3_O_4_ domains via floating self-assembly to fabricate Au/Fe_3_O_4_ Janus nanoparticles without any wet chemical reaction controls. Interestingly, the Au particles in the form of agglomerates were scattered on the Fe_3_O_4_ domains in the form of primary particles owing to mechanical restructuring, and their diameter is given by^18^,^19^,^20^.


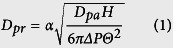


where *D*_pr_ is the diameter of a restructured Au, *α* is the proportionality constant, *H* is the Hamaker constant, *ΔP* is the pressure difference, and *Θ* is the cohesive strength parameter. When the Au agglomerates from the spark ablation reactor were injected into a collison atomizer containing an orifice (0.3 mm diameter), and the agglomerates were subjected to different physical conditions (pressure, density, and velocity), the agglomerates shattered. Thus, the Au agglomerates were redistributed on the Fe_3_O_4_ domains as forms of primary particles (~3 nm) after the orifice via heterogeneous collision between the Au and Fe_3_O_4_, and this induced slight increase in size after the incorporation (Supplementary Fig. S3).

The formation of Au/Fe_3_O_4_ Janus nanoparticles was also verified by UV-vis absorption spectroscopy (Supplementary Fig. S4a). The Au nanoparticles showed an absorption peak at around 525 nm, whereas the spectrum of the Au/Fe_3_O_4_ showed that the incorporation of Au on Fe_3_O_4_ generated an absorption shift from the visible to NIR range^21^, suggesting that the agglomerated Au particles were redistributed or reorganized as Janus nanoparticles via incorporation with Fe_3_O_4_ domains. X-ray diffraction (XRD) patterns (Supplementary Fig. S4b) of the Au, Fe_3_O_4_, and Au/Fe_3_O_4_ samples were analyzed. The six characteristic bands at 30.4° (220), 35.4^o^ (311), 43.2^o^ (400), 53.4^o^ (422), 57.2^o^ (511), and 62.7^o^ (440) were found in the pure Fe_3_O_4_ sample. In the case of Au/Fe_3_O_4_, an additional peak was measured at 38.1^o^ (111), suggesting that the floated Fe_3_O_4_ domains were physically incorporated with face-centered cubic Au particles (well matched with the profile of pure Au particles in Supplementary Fig. S4b) as homogeneous crystallites.

To employ the Janus nanoparticles in MR imaging, the particles were placed in a vibrating sample magnetometer to characterize the magnetic properties at 300 K. Supplementary Fig. S5 shows the paramagnetic characteristics without hysteresis of Fe_3_O_4_ for both the Fe_3_O_4_ and Au/Fe_3_O_4_ samples, corresponding to free coercivity (or remanence). After the magnetic properties were measured, the abilities of the Au/Fe_3_O_4_ Janus nanoparticles for CT-MR dual-mode imaging of a phantom were measured at different mass concentrations (mg mL^−1^ for CT) and concentrations (mM for MR) after the sampled aerosol particles were suspended in water. For measurements of CT contrast ability, the signal intensity was proportional to the particle concentration (inset of Supplementary Fig. S5), which implies that the prepared Au/Fe_3_O_4_ would be available for X-ray imaging applications. To examine MR imaging, an *in vitro T*_2_-weighted MR imaging experiment was further conducted at the same phantom. In the case of MR imaging, the signal intensity was inversely proportional to the particle concentration (with an *r*_2_ of 130.4 mM^−1^ s^−1^, inset of Supplementary Fig. S5). Since the Janus particles are sensitive in both CT and MR imaging, the nanoparticles are expected to have potential for application in CT-MR dual-mode imaging.

**Figure 3:**
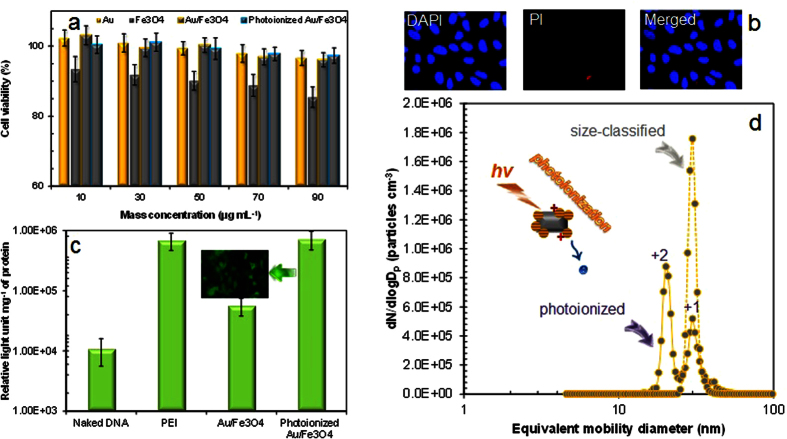
(**a**) *In vitro* cytotoxicity of Au, Fe_3_O_4_, Au/Fe_3_O_4_, and photoionized Au/Fe_3_O_4_ nanoparticles. (**b**) Fluorescence microscope images of HEK 293 cells treated with DAPI and PI. Average cell viability estimations were replicated twice with triplicate repeated measurements. (**c**) Gene-delivery efficiency of photoionized Au/Fe_3_O_4_ nanoparticles compared with PEI-treated and untreated Au/Fe_3_O_4_-gene complexes including naked DNA. EGFP expression for photoionized Au/Fe_3_O_4_ nanoparticles is also shown as inset. (**d**) Charge distributions of Au/Fe_3_O_4_ nanoparticles before and after irradiation with UV light with wavelength of 185-nm.

The sampled nanoparticles on a glass substrate were detached in an ultrasound bath to secure stability for a long-term storage. The dynamic light scattering (DLS) (Nano ZS90, Malvern Instruments, UK) measurements of the photoionized Au/Fe_3_O_4_ particles were additionally performed. The directly gas-phase sampled particles on a glass plate were applied just before [i.e., the injection of the particles on a glass plate in phosphate buffered saline (PBS) solution was performed just before DLS measurements and biological assessments.] required assessments. The results showed that the deviation of hydrodynamic diameter is no larger than 6.1% for the particles, and there are no significant differences between the storage days (1–14 days). This implies that the particles have stability that warrants further investigation. The cytotoxicities of the aerosol-fabricated Au/Fe_3_O_4_ nanoparticles, including individual Au and Fe_3_O_4_ nanoparticles, were tested to verify their biocompatibility using a MTS assay. As shown in Fig. 3a, the cell viabilities were over 96% for the Au/Fe_3_O_4_ and photoionized Au/Fe_3_O_4_ samples at different concentrations from 10 to 90 μg mL^−1^, whereas the viabilities for the Au and Fe_3_O_4_ particles were over 97% and 85%, respectively. To further evaluate the biocompatibility of photoionized Au/Fe_3_O_4_ sample (Fig. 3b), two specific dyes, 4′-6-diamidino-2-phyenylindole (DAPI) (Sigma-Aldrich, US) and propidium iodide (PI) (BD Science, US), were employed to verify viability of cells with the sample. DAPI (blue-350 nm) stains both live and dead cells, while PI (red-488 nm) may only pass through the membranes of dead cells. By examining the fluorescence (IX71, Olympus, US), cell viability was then confirmed, and the results supported the MTS assay that the photoionization did not increase the cytotoxicity of the Au/Fe_3_O_4_ particles. In particular, the cytotoxicity of the Janus particles did not show significant differences compared with individual Au particles, and this implies that the incorporation of Au with Fe_3_O_4_ may even reduce the slightly higher cytotoxicity of the Fe_3_O_4_ nanoparticles. Specifically, enzymatic degradation in cells via catalytic oxidative stress from Fe_3_O_4_ nanoparticles might be somewhat prevented by the presence of the Au particles on the Fe_3_O_4_ domains^22^. This suggests that the Janus particles are not cytotoxic and are likely suitable as nanoplatforms for biomedical applications. To determine the gene-delivery ability of the photoionized Janus particles, the particles were mixed with plasmid DNA to form Au/Fe_3_O_4_-gene complexes and placed in 293 human embryonic kidney cells. The untreated Janus particles showed a higher gene-delivery efficiency than that of naked DNA, and the efficiency was significantly increased when the Janus particles were photoionized, even higher than that of polyethylenimine (PEI)-gene complexes (Fig. 3c). Figure 3c (inset) also shows a visualized enhanced green fluorescent protein (EGFP) distribution in the cells for the photoionized Janus particles, and this provides further evidence of the enhanced gene-delivery via photoionization. In the present work, direct photoionization was employed to create positively charged Au/Fe_3_O_4_ particles, since the photon energy of the employed UV (6.22 eV) is higher than the work function (5.31 eV) of the Au particles that induced electron ejections when the photons absorbed on an Au particle. The enhanced efficiency of the photoionized Janus particles may have been due to the positive charges on the particle surfaces. To verify the electron ejections from the nanoparticles (i.e., positive charges on the particles), we measured the charge distributions of the nanoparticles after the UV irradiation using a tandem differential mobility analyzer (TDMA) system. Figure 3d shows the charge distributions of the particles exposed to UV light with a wavelength of 185-nm. The different peaks appearing in the image represent the different numbers of positive charges on the Janus particles. The ratio between the measured mobilities of two types of nano DMA (NDMA, 3085, TSI, USA) corresponds to the number of charges (*q*) of the photoionized Janus particles, and it is given by^23^.


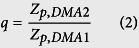


where *Z*_p, DMA 1_ and *Z*_p, DMA 2_ are the measured mobilities using NDMA 1 and 2, respectively. According to the results, the Janus particles were positively charged upon photoionization, and this proves that electrons were ejected from the nanoparticles. The result reveals that the UV irradiation induced positive charges on the particles and that the mean number of charges for the Au/Fe_3_O_4_ particles was 1.44. This also introduced a positive value (+3.4 mV) in zeta potential (measured also using Nano ZS90, Malvern Instruments, UK) of the particles in PBS solution. Supplementary Fig. S6 shows the Au 4*f* X-ray photoelectron spectroscopy (XPS) spectrum of UV-exposed Au/Fe_3_O_4_ nanoparticles. Interestingly, the spectrum also shows a new feature compared with the bulk Au. A well-defined peak appears at 85.2 eV, which is assigned to ionized Au species (Au^*δ*+^), corresponding to the positive charges measured by the TDMA system, whereas there is no additional peak in the untreated Au/Fe_3_O_4_ nanoparticles. Quantitative cellular uptake of photoionized Au/Fe_3_O_4_ nanoparticles (Supplementary Fig. S7) was performed using a fluorescence-activated cell sorting (FACS, BD Biosciences, USA). In the cells, the photoionized particles incorporated with fluorescein isothiocyanate (FITC, as fluorescent tracers, shown in inset of Supplementary Fig. S7) were uptaken in a time-dependent manner. These results suggested further investigation of the photoionized particles.

**Figure 4:**
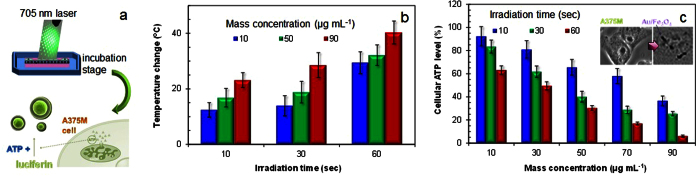
Results for photothermal therapy using Au/Fe_3_O_4_ Janus nanoparticles under 705 nm wavelength laser irradiation. (**a**) Scheme of evaluation process using *in vitro* ATP assay for photothermal therapy. (**b**) Temperature change in Au/Fe_3_O_4_ nanoparticles at different particle concentrations (10–90 μg mL^−1^) by irradiation of 705-nm wavelength laser for durations of 10, 30, and 60 s. (**c**) Photothermal activity to reduce ATP levels for different laser irradiation times (10–60 s) on Au/Fe_3_O_4_ nanoparticles at particle concentrations of 10, 30, 50, 70, and 90 μg mL^−1^.

Au/Fe_3_O_4_ hetero-nanostructures have recently been explored as photoinducers for photothermal cancer cell killing under NIR irradiation^4^. To verify this ability, the Janus nanoparticles were injected into agar gel (as a simulated physiological condition), and they were exposed to a laser with a 705-nm wavelength (Fig. 4a). According to the absorption spectrum (Supplementary Fig. S4a), the laser was selected as the NIR irradiation source to generate heat. The temperature changes under the laser irradiation for durations of 10, 30, and 60 s were measured using an IR thermometer (42545, Extech, USA). In the absence of the Janus particles, no temperature change (Δ*T*, Supporting Information) was found for the same irradiation durations. The temperatures of the agars gels were significantly changed for 10, 30, and 60 s irradiations when the gels contained 10, 50, or 90 μg mL^−1^ of Janus nanoparticles (Fig. 4b). The Δ*T* value was proportional to the particle concentrations, and the value reached 40.3 °C at the highest concentration (90 μg mL^−1^) and longest irradiation time (60 s). From the temperature measurements, it can be concluded that the Janus nanoparticles absorbed the irradiated laser light, and the light was successively converted into thermal energy.

To measure the photothermal activity of the particles for cell killing, an adenosine triphosphate (ATP) assay was employed, since ATP production is related to glycolysis for most cancer cells^24^. A375M cell lines were used to test whether laser exposure can affect ATP production. According to optical microscopy observations, the Janus particles (dark spots, inset of Fig. 4c) were located mostly inside the cell or on the surface of the cell, which confirms the particles’ presence in the cell. No temperature increase in the absence of the Janus particles introduced no significant changes in ATP production, whereas the thermal energy in the presence of the particles obstructed the ATP production of the cells owing to the heat created when the laser irradiated the agar gels. The decrease in ATP production level may have been due to stress gene overexpression (i.e., inducing increasing demands on the ATP cellular pool) via the hyperthermic effect under laser irradiation in the presence of the Janus particles^25^.

**Figure 5:**
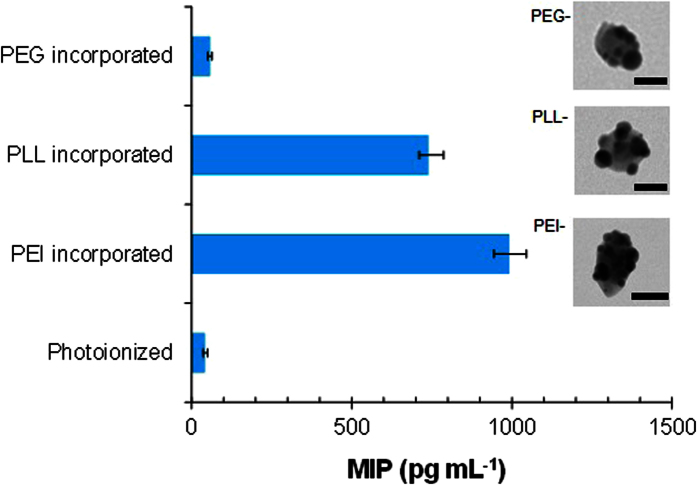
MIP production from LPS-challenged macrophages by adding photoionized and PEI, PLL, and PEG incorporated (2 × 10^−6^ mol dm^−3^) Janus particles. Insets show representative TEM images (scale bar, 20 nm) of the polymer incorporated Janus particles.

Furthermore, another scenario for parenteral applications was considered, where the Janus particles are administered to tissues that attract activated macrophages. In order to suppress inflammatory responses, nanoparticle interaction with biological system has recently been studied for nanoparticulate delivery systems^26^. The results (Fig. 5) show that the photoionized Janus particles could more significantly suppress the macrophage inflammatory protein (MIP) production from lipopolysaccharide (LPS)-challenged macrophages than those from polyethylenimine (PEI) or poly-l-lysine (PLL) incorporated Janus particles (insets of Fig. 5). The smaller MIP productions of the photoionized Janus particles than that from the PEI or PLL incorporated particles indicate that the tendency may be related to the amine content. A similar result from the polyethylene glycol (PEG) incorporated Janus particles (another inset of Fig. 5) further confirms this hypothesis because of no amine groups in PEG.

This study discussed the use of the aerosol route for the fabrication of positively charged Au/Fe_3_O_4_ Janus nanoparticles via the floating self-assembly of Au and Fe_3_O_4_ components and successive photoionization to liberate several electrons from the Janus particles. The fabricated Janus nanoparticles with positive charges were then employed to confirm their biocompatibility and multifunctional abilities for CT-MR dual-mode imaging, gene-delivery, and photothermal therapy without significant increases in cytotoxicity or changes in morphology. Even though all previous Au-based hetero-nanostructures employed polymeric or macromolecular organic layers on which nanostructures were loaded, this study suggests a new possibility of fabricating multibiofunctional nanoplatforms with the minimized inflammatory responses without the use of multiple wet chemical processes and organic compounds. This original, fundamental strategy of unusual urgency and significance may appeal to a broad, general audience in biomedical applications.

## Additional Information

**How to cite this article**: Byeon, J. H. and Park, J. H. Use of aerosol route to fabricate positively charged Au/Fe_3_O_4_ Janus nanoparticles as multifunctional nanoplatforms. *Sci. Rep*. **6**, 35104; doi: 10.1038/srep35104 (2016).

## Supplementary Material

Supplementary Information

## References

[b1] Wang C. . Recent progress in syntheses and applications of dumbbell-like nanoparticles. Adv. Mater. 21, 3045–3052 (2009). 20011128 10.1002/adma.200900320 PMC2792936

[b2] Xu C. . Au-Fe_3_O_4_ dumbbell nanoparticles as dual-functional probes. Angew. Chem.-Int. Ed. 47, 173–176 (2008). 10.1002/anie.200704392 PMC2692410 17992677

[b3] Zhai Y. . Dual-functional Au-Fe_3_O_4_ dumbbell nanoparticles for sensitive and selective turn-on fluorescent detection of cyanide based on the inner filter effect. Chem. Commun. 47, 8268–8270 (2011). 10.1039/c1cc13149d 21695338

[b4] Ren J. . Facile synthesis of superparamagnetic Fe_3_O_4_@Au nanoparticles for photothermal destruction of cancer cells. Chem. Commun. 47, 11692–11694 (2011). 10.1039/c1cc15528h 21952492

[b5] Zijlstra P. & Orrit M. Single metal nanoparticles: optical detection, spectroscopy and applications. Rep. Prog. Phys. 74, 106401–106456 (2011).

[b6] Neumann O. . Solar vapor generation enabled by nanoparticles. ACS Nano 7, 42–49 (2013). 23157159 10.1021/nn304948h

[b7] Pissuwan D., Valenzueka S. M. & Cortie M. B. Therapeutic possibilities of plasmonically heated gold nanoparticles. Trends Biotechnol. 24, 62–67 (2006). 16380179 10.1016/j.tibtech.2005.12.004

[b8] Huang C. . Trapping iron oxide into hollow gold nanoparticles. Nanoscale Res. Lett. 6, 1–5 (2011). 10.1007/s11671-010-9792-x PMC3211847 27502665

[b9] Smolensky E. D. . Fe_3_O_4_@organic@Au: core-shell nanocomposites with high saturation magnetisation as magnetoplasmonic MRI contrast agents. Chem. Commun. 47, 2149–2151 (2011). 10.1039/c0cc03746j PMC3077948 21165501

[b10] Wang G. . Au nanocage functionalized with ultra-small Fe_3_O_4_ nanoparticles for targeting *T*_1_-*T*_2_ dual MRI and CT imaging of tumor. Sci. Rep. 6, 28258 (2016). 27312564 10.1038/srep28258 PMC4911575

[b11] Zhao H. Y. . Synthesis and application of strawberry-like Fe_3_O_4_-Au nanoparticles as CT-MR dual-modality contrast agents in accurate detection of the progressive liver disease. Biomaterials 51, 194–207 (2015). 25771010 10.1016/j.biomaterials.2015.02.019

[b12] Huang J. . Rational design and synthesis of *γ*Fe_2_O_3_@Au magnetic gold nanoflowers for efficient cancer theranostics. Adv. Mater. 27, 5049–5056 (2015). 26198387 10.1002/adma.201501942

[b13] Beveridge J. S. . Purification and magnetic interrogation of hybrid Au-Fe_3_O_4_ and FePt-Fe_3_O_4_ nanoparticles. Angew. Chem.-Int. Ed. 50, 9875–9879 (2011). 10.1002/anie.201104829 21898742

[b14] Salado J. . Functionalized Fe_3_O_4_@Au superparamagnetic nanoparticles: *in vitro* bioactivity. Nanotechnology 23, 315102 (2012). 22802157 10.1088/0957-4484/23/31/315102

[b15] Byeon J. H., Park J. H. & Hwang J. Spark generation of monometallic and bimetallic aerosol nanoparticles. J. Aerosol Sci. 39, 888–896 (2008).

[b16] Yu H. . Dumbbell-like bifunctional Au-Fe_3_O_4_ nanoparticles. Nano Lett. 5, 379–382 (2005). 15794629 10.1021/nl047955q

[b17] Wei Y. . Synthesis, shape control, and optical properties of hybrid Au/Fe_3_O_4_ “nanoflowers”. Small 4, 1635–1639 (2008). 18636405 10.1002/smll.200800511

[b18] Byeon J. H. & Roberts J. T. Aerosol based fabrication of biocompatible organic-inorganic nanocomposites. ACS Appl. Mater. Interfaces 4, 2693–2698 (2012). 22509789 10.1021/am300337c

[b19] Byeon J. H. & Kim J.-W. Aerosol fabrication of thermosensitive nanogels and *in situ* hybridization with iron nanoparticles. Appl. Phys. Lett. 101, 023117 (2012).

[b20] Byeon J. H. & Roberts J. T. Aerosol based fabrication of thiol-capped gold nanoparticles and their application for gene transfection. Chem. Mater. 24, 3544–3549 (2012).

[b21] Lou L. . Facile methods for synthesis of core-shell structured and heterostructured Fe_3_O_4_@Au nanocomposites. Appl Surf. Sci. 258, 8521–8526 (2012).

[b22] Wang Y. . A simple method to construct bifunctional Fe_3_O_4_/Au hybrid nanostructures and tune their optical properties in the near-infrared region. J. Phys. Chem. C 114, 4297–4301 (2010).

[b23] Byeon J. H. . Charge distributions of aerosol dioctyl sebacate particles charged in a dielectric barrier discharger. J. Aerosol Sci. 39, 460–466 (2008).

[b24] Huang H. . L-carnitine is an endogenous HDAC inhibitor selectively inhibiting cancer cell growth *in vivo* and *in vitro*. PLoS One 7, e49062 (2012). 23139833 10.1371/journal.pone.0049062 PMC3489732

[b25] Tedja R. . Biological impacts of TiO_2_ on human lung cell lines A549 and H1299: particle size distribution effects. J. Nanopart. Res. 13, 3801–3813 (2011).

[b26] Dobrovolskaia M. A. . Preclinical studies to understand nanoparticle interaction with the immune system and its potential effects on nanoparticle biodistribution. Mol. Pharmaceut. 5, 487–495 (2008). 10.1021/mp800032f PMC2613572 18510338

