# Supplementary material for: Use of aerosol route to fabricate positively charged Au/Fe3O4 Janus nanoparticles as multifunctional nanoplatforms
Source: Sci Rep. 2016 Oct 7;6:35104. doi: 10.1038/srep35104 (PMC5054399; doi:10.1038/srep35104)
Supplement: Supplementary Information [file srep35104-s1.pdf]

Supplementary Information for Publication

Use of aerosol route to fabricate positively charged Au/Fe_3O_4 Janus nanoparticles as multifunctional nanoplatforms

Jeong Hoon Byeon[1] & *Jae Hong Park*[2]

[1]School of Mechanical Engineering, Yeungnam University, Gyeongsan 38541, Republic of Korea

[2]School of Health Sciences, Purdue University, IN 47907, United States

Correspondence and requests for materials should be addressed to J.H.B (email: postjb@yu.ac.kr) or J.H.P. (email: park895@purdue.edu)

METHODS

- *Gas-liquid hybrid synthesis of positively charged Au/Fe_3O_4 Janus nanoparticles*

As shown in **Fig. S1**, a spark discharge was used to generate Au nanoparticles in the gas-phase, and the particle-laden flow was employed as the operating gas for atomizing the Fe_3O_4 solution. A spark discharge has been used to produce a variety of metallic, carbonaceous, and other composite materials with nanoscale dimensions at ambient temperatures and pressures.[S1-S5] For the preparation of the Fe_3O_4 nanoparticles, Solution 1 was a mixture solution of 0.074 g $FeCl_3 \cdot 6H_2O$ (Sigma-Aldrich, US) and 0.026 g $FeCl_2 \cdot 6H_2O$ (Sigma-Aldrich, US) in 30 mL ethanol, and Solution 2 was a mixture solution of 25 mL ethanol and 5 mL ammonia (28-30%). Solutions 1 and 2 were injected drop by drop with the aid of a peristaltic pump (323Du/MC4, Watson-Marlow Bredel Pump, US) at constant rates of 6 and 4 mL min^{-1}, respectively. Solutions 1 and 2 were mixed in a flask and then an ultrasonic probe (VCX 750, 13 mm titanium alloy horn, 20 kHz, Sonics & Materials Inc., US) was immersed into the mixture solution. The probe acted as an ultrasound irradiator (10 W mL^{-1} input power density) and the active part of the probe was the planar circular surface, of area 1.3 cm^2, at the bottom of the probe.[S6] The Au nanoparticles passed over the atomizer orifice, where they mixed with atomized Fe_3O_4 droplets to form hybrid droplets. The droplets then passed through a tubular chamber with 185 nm UV irradiation at an intensity of 0.14 J m^{-2} s^{-1} to induce photoionization after solvent extraction of the droplets *via* a denuder containing activated carbons and silica gels.

- *In vitro computed tomography (CT) and magnetic resonance imaging (MRI)*

Aqueous dispersions of Au/Fe_3O_4 nanoparticles with different mass concentrations were examined with a 9.4 T small animal MRI scanner (Bruker) to evaluate the contrast enhancement effect. T_2-weighted imaging was performed using an inversion recovery gradient echo sequence with TE = 4 ms, a slice thickness of 0.5 mm, an field of view of 3 × 3 cm, and a matrix size of 128 × 128.

CT scans were performed using GE Light Speed VCT imaging system (GE Medical Systems) operated at 100 kV and 80 mA, with a slice thickness of 0.625 mm. Dispersions with different mass concentrations were prepared in 2.0 mL Eppendorf tubes and placed in a self-designed scanning holder. Contrast

enhancement was determined in Hounsfield units for each sample.

- *Cell viability and gene-delivery*

The cytotoxicity of the prepared Janus nanoparticles was evaluated using 293 human embryonic kidney (HEK) cells by the MTS, 3-(4,5-dimethyl-thiazol-2-yl)-5-(3-carboxymethoxyphenyl)-2-(4-sulfophenyl)2H-tetrazolium, assay. The cells were cultured in 200 mL Dulbecco's modified eagle medium (DMEM, Carlsbad, US) supplemented with 10% fetal bovine serum (FBS) at 37°C, 5% CO_2, and 95% relative humidity. The cells were seeded in a 96-well microtiter plate (Nunc, Germany) at densities of 1×10^5 cells $well^{-1}$. After 24 h, the culture media were replaced with serum-supplemented culture media containing the modified chitosans (1mg mL^{-1}), and the cells were incubated for 24 h. Then, 30 μL of the MTS reagent was added to each well. The cells were incubated for an additional 2 h. The absorbance was then measured using a microplate reader (Spectra Plus, TECAN, Switzerland) at a wavelength of 490 nm. The cell viability (%) was compared with that of the untreated control cell in media without Janus nanoparticles and calculated with $[A]_{test}$:$[A]_{control} \times 100\%$, where $[A]_{test}$ is the absorbance of the wells with Janus nanoparticles and $[A]_{control}$ is the absorbance of the control wells.

The HEK cells were seeded in 24-well plates at a density of 1×10^6 cells $well^{-1}$ in 1 mL of complete DMEM medium supplement with 10% FBS at 37°C, 5% CO_2, and 95% relative humidity, one night before transfection. The culture medium was replaced with serum free DMEM medium, and transfection complexes were added to the cells. The cells were incubated with the transfection complexes at 37°C for an additional 24 h after the medium was replaced by fresh complete medium. After incubation for 24 h, the medium was aspirated and washed with phosphate-buffered saline. The cells were trypsinized and then the transfection results were measured by fluorescence activated cell sorting. The green fluorescent protein expression of the Janus nanoparticles in the cells was observed with a fluorescent microscope (Nikon Eclipse TE2000-S, US). The luciferase activity was measured with a luminometer (9100-102, Turner Biosystems, US). The final luciferase activity was expressed in relative light units (RLU) per mg of protein.

All experiments were performed in triplicate, and the results were reported as average values and

standard deviations. The Student's t-test was performed to determine statistical significance between untreated and treated groups. $P < 0.05$ was regarded as statistically significant.

- *Measurements in surface charge of the nanoparticles*

The TDMA system consisted of two NDMAs (3085, TSI, US), NDMA 1 and 2, and a condensation particle counter (CPC, 3776, TSI, US). Even though DMAs have mostly been employed for the environmental sciences, a DMA was recently employed to classify cationic polymer nanoparticles for biomedical applications.[S7] NDMA 1 and 2 were placed before and after a UV chamber, respectively, which contained a UV lamp (UVP, UK) with a wavelength of 185 nm. NDMA 1 was operated as an electrostatic particle classifying system, which was operated at a chosen fixed voltage provided by a direct current power supply (205B, Bertan, US) to extract the particles of equivalent electrical mobility. The particles exiting NDMA 1 (all with equivalent electrical mobility) passed through a serial system consisting of an aerosol charge neutralizer (4810, HCT, Korea) and a cylindrical electrostatic precipitator to form uncharged monodisperse particles (as 20 nm), and finally the particles were then fed into the UV chamber. The particles from the UV chamber were finally scanned by NDMA 2 to measure the charge distribution corresponding to the initially selected mobility diameter.

- *Cellular uptake*

To quantitatively measure cellular uptake, the HEK cells (1×10^5 cells per well) were seeded in 12 well plates, and incubated for 48 h. The cells were treated with FITC incorporated photoionized Au/Fe_3O_4 particles at a concentration of 5 μg mL^{-1} in a humidified incubator with 5% CO_2 atmosphere at 37°C. After 60 min incubation, the cells were washed with PBS solution and harvested. The cells were then dispersed in 1.0 mL of PBS solution for flow cytometric measurements using a FACS Calibur flow cytometer (BD Biosciences, US).

- *Photothermal therapy*

Synthesized Au/Fe_3O_4 nanoparticles were evenly dispersed in 2% agar at concentrations of 10, 30, 50, 70, and 90 μg mL^{-1}. The gels were formed in shallow, 35 mm diameter plastic petri dishes. For exposure, the gel phantom samples at room temparature were exposed to a 705 nm continuous wave laser beam (40

mW, *i.e.* 4.12 W cm^{-2} in power density) emitted by a solid state laser system (HL7001MG, Opnext, Japan). The gel samples were positioned in the laser beam and irradiated by the beam for fixed durations of 10, 30, and 60 s. In order to evaluate the application to photothermal therapy, ATP assay was further employed, which is based on the highly sensitive firefly reaction to determine the level of cellular ATP as a surrogate marker for the number of live cells.[S8] After a 24 h incubation with Au/Fe_3O_4 nanoparticles, the cells were washed three times with Hank's buffered salt solution and 0.1 mL of CellTiter-Glo Luminescent (Promega, US) assay reagent was added to each well and the plate was then mixed using an orbital shaker for 2 min, followed by 10 min incubation to stabilize the luminescence signals. Luminescence was read using the luminometer.

- *Macrophage Inflammatory Protein (MIP) Production*

Peritoneal macrophages were seeded in 24-well plates at a density of 10^5 cells per well in 1 mL of medium. After overnight incubation, 0.1 mL of the Janus particle solution was injected to each well to set the particle concentraion in medium to 2 mg mL^{-1}. For comparison purposes, 0.1 mL of polyethyleneimine (PEI, 765090, Sigma-Aldrich, US), poly-l-lysine (PLL, P4707, Sigma-Aldrich, US), or polyethylene glycol (PEG, 81188, Sigma-Aldrich, US) was injected in lieu of the Janus particle solutions. After 24 h incubation, the culture media were centrifuged at 2000 rpm for 10 min to separate supernatants. Macrophages were challenged by adding lipopolysaccharide (LPS) to the media in the final concentration of 1 μg mL^{-1} shortly before the comparisons. Enzyme-linked immunosorbent assay (ELISA) was performed to determine the MIP levels using MIP-2 ELISA kit (R&D Systems, US). The supernatants collected from LPS-challenged macrophages was always diluted 10 times prior to the analysis. The differences were considered significant for $p < 0.01$.

Fig. S1

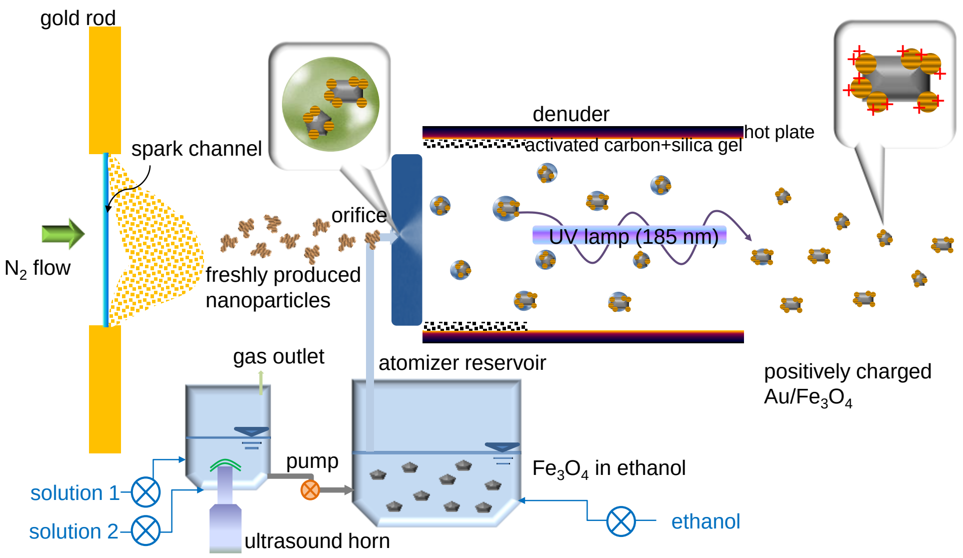

Single-pass aerosol route to fabricate positively charged Au/Fe_3O_4 Janus nanoparticles.

Fig. S2

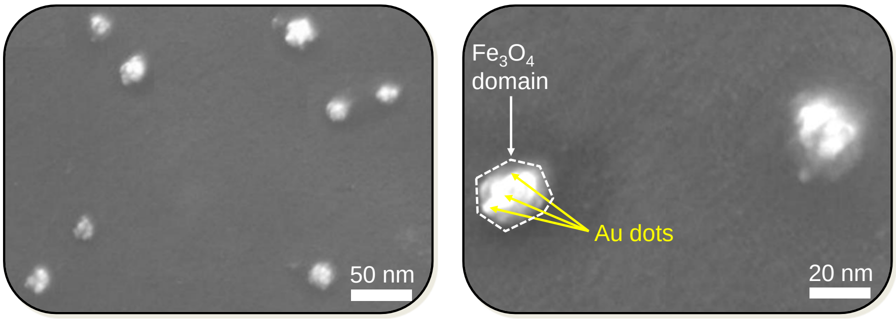

Low and high-magnitude SEM images of Au/Fe_3O_4 nanoparticles.

Fig. S3

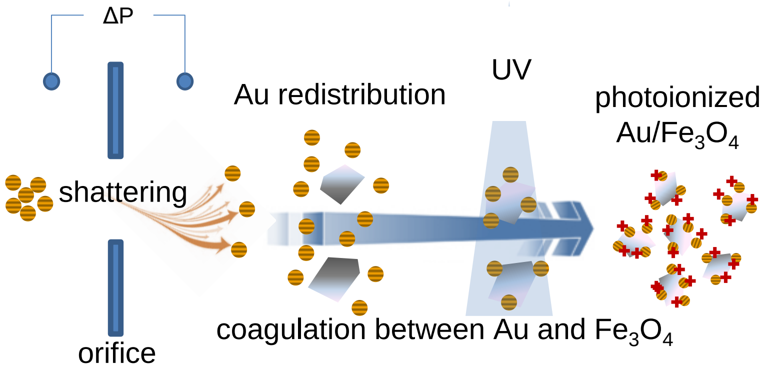

Mechanism of incorporation between Au agglomerates and Fe_3O_4 domains in the gas-phase. The de-agglomarated Au particles are deposited on Fe_3O_4 domains via heterogeneous collision between them.

Fig. S4

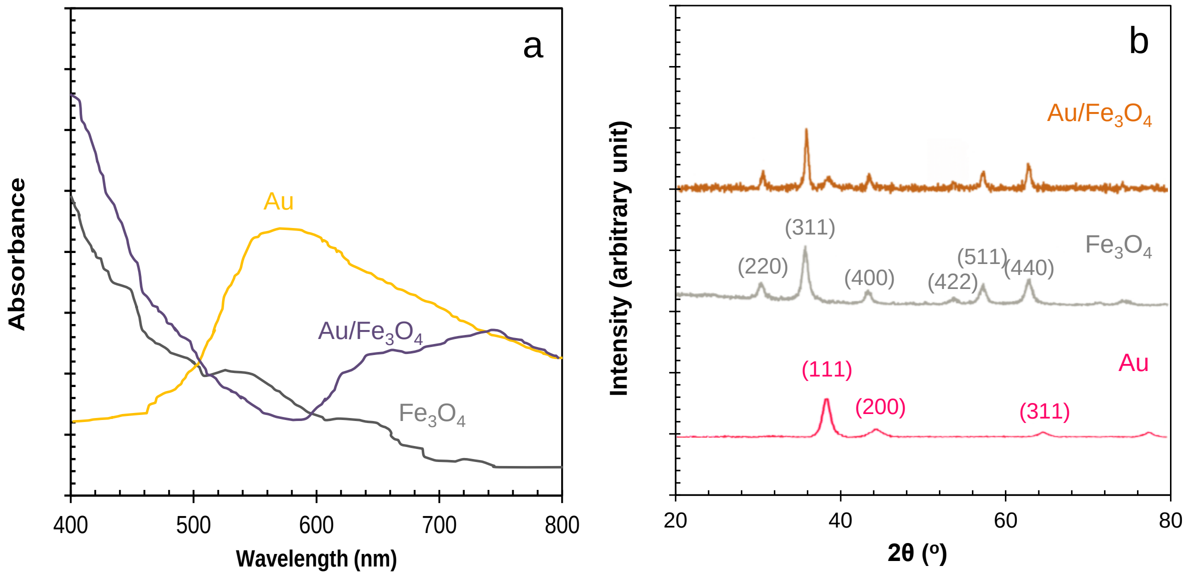

(a) UV-vis absorption spectroscopy (330, Perkin-Elmer, US) of Au, Fe_3O_4, and Au/Fe_3O_4 nanoparticles. (b) XRD (RINT-2100, Rigaku, Japan) patterns of Au, Fe_3O_4 and Au/Fe_3O_4 nanoparticles.

The broadening of the absorption peak for the Au/Fe_3O_4 may be explained by Mie theory, which is related with an environmental change, such as in the dielectric properties, around the Au particles. This can cause an absorbance shift when Au particles are redistributed on Fe_3O_4 domains as Janus nanoparticles (i.e., NIR responsive).[S9] Additionally, there was decreased reflectance, which might have been due to Fe_3O_4 incorporation (i.e., dilution effect) with Au particles, since Fe_3O_4 domains did not show reflectance at the same measurement condition.

The six characteristic bands at 30.4^o, 35.4^o, 43.2^o, 53.4^o, 57.2^o, and 62.7^o were found in the Fe_3O_4 sample, which are attributable to the Fe_3O_4 inverse spine planes of (220), (311), (400), (422), (511), and (440) (JCPDS 19-0629), respectively. In the case of Au/Fe_3O_4, on the other hand, three additional distinct peaks were measured at 38.1^o, 44.3^o, and 64.6^o [corresponding to the (111), (200), and (311) planes of fcc Au, JCPDS 04-0784],[S10] evidencing the attachment of Au particles on the Fe_3O_4 domains to create Au/Fe_3O_4 hetero-nanostructures.

Fig. S5

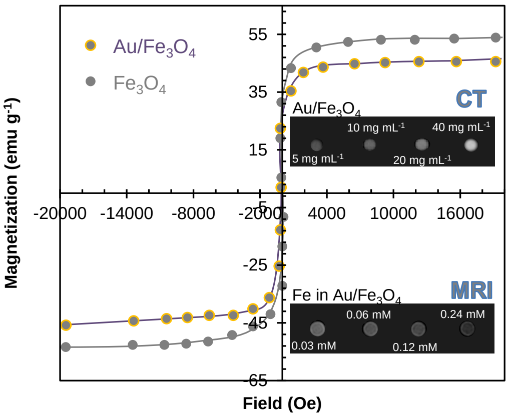

Magnetic properties (7404, Lake Shore Cryotronics, US) of Fe_3O_4 and Au/Fe_3O_4 nanoparticles including CT and T_2-weighted MRI images (insets) at the phantom with various mass concentrations of Au/Fe_3O_4 nanoparticles.

In the case of Au/Fe_3O_4, lower saturation magnetizations were detected than those from the Fe_3O_4 because the Au could contribute to the diamagnetization of the Fe_3O_4. Nevertheless, the magnetization values of Au/Fe_3O_4 were still available to apply magnetic separation applications (i.e., MR imaging in this study).

Fig. S6

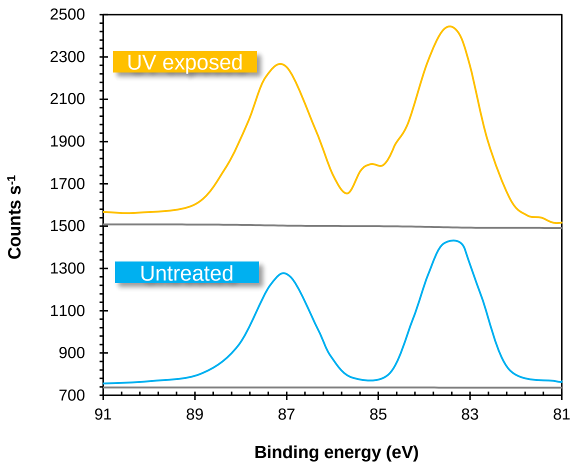

Au 4*f* XPS spectra of untreated and UV-exposed Au/Fe_3O_4 nanoparticles.

The peaks at 83.4 eV and 87.1 eV were assigned to the binding energies of Au $4f_{7/2}$ and Au $4f_{5/2}$, which can be assigned to metallic Au. The slight difference in binding energy between the present and bulk Au assigned to 83.8 eV for Au $4f_{7/2}$ and 87.5 eV for Au $4f_{5/2}$ may have been due to the perturbed electronic state/movement in the Au atomic orbit owing to UV irradiation.

Fig. S7

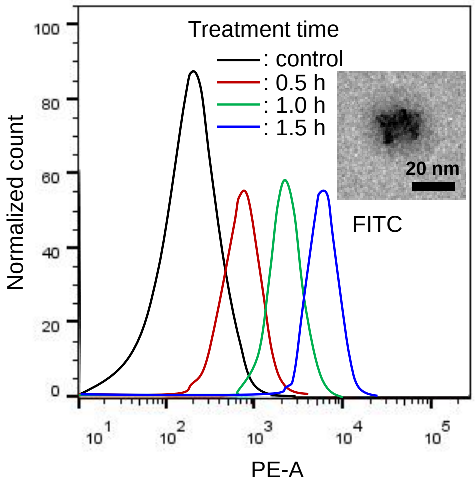

FACS measurements for quantitative cellular uptake analyses of photoionized Au/Fe_3O_4 nanoparticles incorporated with FITC (inset, as fluorescent tracers) in a time-dependent manner.

SPR HEATING

The unique incorporation of Au and Fe_3O_4 components could introduce NIR absorption to convert photon energy to thermal energy, which is suitable for destroying cancer cells by heat. The temperature change via SPR heating can be analytically estimated, and it is given by[S11]

$$\Delta T\left(D_p\right)=\frac{V_p P_{abs}}{2\pi k_0 D_p} \tag{S1}$$

where D_p is the particle diameter, V_p is the particle volume, k_0 is the thermal conductivity, and P_{abs} is the light-induced heating. Based on this photothermal conversion of the Janus particles upon 705-nm excitation, in vitro photothermal therapy using photoionized Janus particles was investigated.

SUPPLEMENTARY TABLE

Table S1 A summary of the size distributions of spark-produced Au, collison atomized Fe_3O_4, and their incorporated structure (Au/Fe_3O_4) from aerosol route

Case	GMD (nm)	GSD (-)	TNC (× 10^6 particles cm^{-3})
Au	19.7	1.28	3.45
Fe_3O_4	20.2	1.46	3.22
Au/Fe_3O_4	24.7	1.49	4.10

SUPPLEMENTARY REFERENCES

S1. Byeon, J. H., Park, J. H. & Hwang, J. Spark generation of monometallic and bimetallic aerosol nanoparticles. *J. Aerosol Sci.* **39**, 888-896 (2008).

S2. Byeon, J. H. *et al.* Removal of volatile organic compounds by spark generated carbon aerosol particles. *Carbon* **44**, 2106-2108 (2006).

S3. Byeon, J. H. *et al.* Ambient spark generation to synthesize carbon-encapsulated metal nanoparticles in continuous aerosol manner. *Nanoscale* **1**, 339-343 (2009).

S4. Byeon, J. H. & Kim, J.-W. Production of carbonaceous nanostructures from a silver-carbon ambient spark. *Appl. Phys. Lett.* **96**, 153102 (2010).

S5. Byeon, J. H. & Kim, J.-W. Morphology and structure of aerosol carbon-encapsulated metal nanoparticles from various ambient metal-carbon spark discharges. *ACS Appl. Mater. Interfaces* **2**, 947-951 (2010).

S6. Byeon, J. H. & Kim, Y.-W. A novel polyol method to synthesize colloidal silver nanoparticles by ultrasonic irradiation. *Ultson. Sonochem.* **19**, 209-215 (2012).

S7. Byeon, J. H., Kim, H.-K. & Roberts, J. T. Monodisperse poly(lactide-co-glycolic acid)-based nanocarriers for gene transfection. *Macromol. Rapid Commun.* **33**, 1840-1844 (2012).

S8. Ulukaya, E. *et al.* Cell death-inducing effect of novel palladium (II) and platinum (II) complexes on non-small cell lung cancer cells in vitro. *J. Cancer Res. Clin. Oncol.* **137**, 1425-1434 (2011).

S9. Lou, L. *et al.* Facile methods for synthesis of core-shell structured and heterostructured Fe_3O_4@Au nanocomposites. *Appl Surf. Sci.* **258**, 8521-8526 (2012).

S10. Wang, Y. *et al.* A simple method to construct bifunctional Fe_3O_4/Au hybrid nanostructures and tune their optical properties in the near-infrared region. *J. Phys. Chem. C* **114**, 4297-4301 (2010).

S11. Neumann, O. *et al.* Solar vapor generation enabled by nanoparticles. *ACS Nano* **7**, 42-49 (2013).
